# Unraveling the Determinants of Protrusion Formation

**DOI:** 10.1155/2012/402916

**Published:** 2012-03-01

**Authors:** Mita Varghese, Peter Gorsevski, Marilyn L. Cayer, Nancy S. Boudreau, Carol A. Heckman

**Affiliations:** ^1^Department of Biological Sciences, Bowling Green State University, Bowling Green, OH 43403-0212, USA; ^2^School of Earth, Environment and Society, Bowling Green State University, Bowling Green, OH 43403-0212, USA; ^3^Center for Microscopy and Microanalysis, Bowling Green State University, Bowling Green, OH 43403-0212, USA; ^4^Department of Applied Statistics and Operations Research, Bowling Green State University, Bowling Green, OH 43403-0212, USA

## Abstract

A computerized morphometric classification technique based on latent factors reveals major protrusion classes: factors 4, 5, and 7. Previous work showed that factor 4 represented filopodia, 5 the distribution of lamellar cytoplasm, and 7 a blunt protrusion. We explore the relationship of focal contact (FC) characteristics and their integrated actin cables to factors values. The results show that FC maturation/cytoskeletal integration affects factor 5, because FC elongation/integration was correlated with its values. On the contrary, 7 values decreased with maturation, so cable or FC size or their integration must be restricted to form these protrusions. Where integration did occur, the cables showed distinctive size and orientation, as indicated by correlation of 7 values with FC shape. Results obtained with myosin inhibitors support the interpretation that a central, isometric, contractile network puts constraints on both factor 5 and 7 protrusions. We conclude that cells establish functional domains by rearranging the cytoskeleton.

## 1. Introduction

Cell migration is integral to embryonic development, wound healing, immune responses, and tissue differentiation. Cells in motion generate two major types of protrusive structures, lamellipodia and filopodia, which have characteristically different actin polymerization machinery and are regulated by different signaling pathways. These protrusive structures are regulated through actin filament organizers, such as nucleation factors and bundling proteins, which are in turn regulated by signaling molecules, RhoA, Rac, and/or Cdc42 [1–5]. In the lamellipodium, a dense network of short, branched actin filaments is formed driven by Arp2/3 complex activation. These filaments are elongated, cross-linked at their intersections, and some are capped on the barbed end. In contrast, filopodia are thin, tapering, finger-like protrusions that contain a bundle of parallel actin filaments [6, 7].

Universally, cell motility involves a highly orchestrated cycle of steps in which forces in the actomyosin cytoskeleton are spatially and temporally coordinated with extracellular adhesion [8, 9]. Protrusion of the leading edge is thought to be driven by actin polymerization occurring in two distinct zones, the lamellipodium, and the lamellum [10–12]. The actin network beneath the membrane undergoes rapid retrograde flow due to polymerization at the barbed end of the filament [11, 13–15]. In contrast, the lamellum has bundles of actin filaments that undergo a slower retrograde movement [16, 17], reviewed in [18]. In the lamellipodium-lamellum interface, known as the transition zone, the dendritic network of actin is partially depolymerized and reorganized into bundles [11, 19–21]. Although leading-edge protrusion is widely thought to be dependent on the balance of actin assembly and retrograde flow produced by actin polymerization, the role of myosin in the advance of the leading edge is disputed [20, 22–24].

 Protrusions are stabilized by adhesive protein complexes that link the actin cytoskeleton via integrins to the underlying extracellular matrix proteins. Previous work established that nascent FCs form as small, dot-like structures located in a meshwork of short, branched actin filaments at the periphery of the lamellipodia (reviewed in [25–28]). These FCs turn over in ~60 seconds. In neuronal growth cones, they are known as point contacts [29] and are regulated by coordinated Rho and Rac GTPase activity to stabilize protrusions and neurite outgrowths [30]. In nonneuronal cells, small FCs are called punctate FCs [31] or Rac-induced FCs [32]. The nascent FCs may mature to larger, elongated adhesions called focal adhesions (FAs). FCs form immediately behind the leading edge, while FAs reside slightly further back from the edge and are concentrated at the lamellipodium—lamellum interface. The FAs persist for several minutes [19, 32–34]. Actomyosin contraction has been implicated in the integration of FCs or FAs with stress fibers, as shown by direct observations [35, 36], reviewed in [37]. A contractile network extends from FAs at the lamellipodial base to near the nucleus and is composed of actomyosin II networks and bundles in the lamella, transition zone, and central cell area [10, 16]. Traction forces are generated between strong FAs at the front and the weaker FAs in the rear, which detach [38, 39] and thereby move the cell body forward in the final step of migration.

Nascent adhesions either mature or disassemble when they encounter the zone of depolymerizing actin at the junction of the lamellipodium and lamellum. Adhesion turnover in this region is therefore coincident with actin severing and the disassembly of branched actin structures. Nascent adhesions can mature into FAs coincident with periodic or occasional pauses of the forward movement of the leading edge. These pauses correlate with, or depend on, myosin II-dependent contractile events [23, 40–43]. A sequential mechanism coupled to myosin II activity or tension exerted on adhesions may be important in determining the balance between adhesion disassembly and maturation. It has been observed that force application induced FC growth, whereas inhibition of actomyosin contractility decreased FC size [31, 44, 45]. Myosin II is also an endpoint of the pathways regulated by Rho GTPases, which are downstream hubs for migration-related signaling pathways [46–49]. Studies have shown that Rac and Cdc42 prevented differentiation into large FAs whereas Rho promoted the enlargement of FCs [32, 42]. Likewise, the myosin II inhibitor, blebbistatin, prevented adhesion maturation and greatly increased nascent adhesions. Conversely, myosin IIA overexpression in CHO cells inhibited leading edge protrusion and increased nascent adhesion maturation to FAs [42]. Initial adhesion assembly is mechanistically and kinetically linked to actin polymerization in the lamellipodium, whereas, if myosin II activity and tension are exerted on actin in the lamellum, it contributes to the maturation of newly formed adhesions to FAs [35, 42, 43, 50–52].

 The current understanding of the regulation of the actin filament, actomyosin contraction, and FC dynamics is as yet insufficient to explain the formation of different protrusion types. Traditional biochemical methods and visualization of selected molecules by microscopy have supplied the basic information needed to elucidate the regulation of cell shape features. Our laboratory has addressed this problem by quantification and classification of features, based on a sample of the mass per area throughout the peripheral cytoplasm of the cell. Beginning with single cell interference images of cell lines that became tumorigenic when maintained over a long-time course in vitro, primary shape variables were extracted that distinguished the normal and transformed cells [53–57]. From 102 primary mathematical variables, the common changes in variance could be analyzed by latent factor extraction in Statistical Analysis Software (SAS). Factor analysis yielded 20 latent factors, which proved to have a one-to-one correspondence to cell features and could be used to distinguish nontumorigenic and tumorigenic cell phenotypes. Of the 20 factors, four were based predominantly upon variables describing the cell edge and therefore corresponded to protrusions as discussed elsewhere [57, 58]. Factor 4 reflected the size and frequency of filopodia or microspikes. The loss of filopodia represented the single greatest source of transformation-related variability. Factor 7 described a feature that was broader and bulkier than filopodia. Of the additional features, factor 5 values were representative of long, sprawling protrusions or deep indentations, that is, centrifugal mass displacement. Finally, factor 16 represented tapering projections larger than filopodia and anchored deeper in the cytoplasm [57, 58].

It is now well established that, of Rho-GTPase family members, RhoA activates the formation of actin stress-fiber bundles and their associated FAs, whereas Rac and Cdc42 regulated lamellipodia and filopodia [2]. It was found earlier that p21-activated kinase (PAK) recruitment to the FC is indispensable to the formation of both factor 4 and factor 7 protrusions. A comparison of FCs in cells expressing PAK in the presence or absence of PAK kinase inhibitor domain (PAK^83−149^) suggested that PAK inhibition enlarged FCs without affecting the prevalence of either protrusion [59]. In the neuronal growth cone, filopodia are organized by a basal organelle, the focal actin ring, and show FCs at their tip, midregion, and base. The actin focal ring is thought to attach actin filaments to the basal FC and thereby facilitate tension development and filopodial emergence [60, 61]. Although the organelle has not been reported in nonneuronal cells, we speculate that the FCs of filopodia would still differ qualitatively from those in other protrusions and, in particular, the factor 7 features. In the current research, we explored the relationship between the characteristics of FCs and the feature types themselves. Thus, we will test the implication that FCs with specific characteristics can favor the formation of a particular feature type. To understand the roles of Rho-kinase (ROCK) and myosin light chain (MLC) kinase for the contraction and organization of stress fibers in either filopodia or other features, we treated rat tracheal epithelial cells with MLCK and Rho-kinase inhibitors and analyzed the cell phenotypes. In smooth muscle cells, ROCK activation stimulated contraction [62], while dominant-acting mutants of ROCK induced stress fibers and focal adhesions [63–66]. Conversely, ROCK inhibition released stress fibers from their attachments to FCs [67].

The approach we use to solve cell shape phenotypes appears to have two important applications in the field of cell adhesion and motility. Since the protrusion types we describe in epithelial cells are more numerous than those described on a subjective basis by previous investigators, it is possible that different protrusions are classed together in the previous work. This fact would go far toward explaining the lack of agreement about the role of myosin in lamellipodial extension and retraction. If the two protrusion types are lumped together, but only one employs myosin for extension or retraction of the cell edge, this would lead to wildly divergent observations. If so, the protrusion with less requirement for contractility would be the factor 7 feature described in the current studies. Secondly, coherent models of how FCs are initiated and assembled have been slow to emerge from experimental work. Even work at the forefront of the field has failed to clarify the processes of FC maturation and disassembly. The current results suggest that cell cytoplasm is partitioned in domains. If extension of each type of protrusion is favored in a specific domain, identifying determinants of the domains would accelerate progress on these problems. It is to be hoped that a better understanding of feature properties will hasten the time when theories explaining the mechanisms of FC maturation and turnover could be put forward.

## 2. Materials and Methods

### 2.1. Cell Culture and Chemical Treatment

The 1000 W line was generated by treatment of a heterotopic tracheal transplant from a Fisher rat with 7, 12-dimethylbenz(a)anthracene. The line was maintained under routine culture conditions in Waymouth's medium containing 10% fetal bovine serum, supplemented with 0.1 *μ*g/mL insulin and 0.1 *μ*g/mL hydrocortisone [54, 68, 69]. For experiments, cells were subcultured into 35 mm plastic Petri dishes or Lab-Tek chamber slides with reactive surfaces (RS) as previously described [70]. At 24 hours after plating, the medium was changed to serum-free and cells were transfected with engineered genes (see plasmids). GFP paxillin along with the construct of interest was mixed with Lipofectamine 2000 reagent and put into the culture for 9-10 h, according to the manufacturer's protocol (Invitrogen, Carlsbad, CA). Cells were left for 6 h in serum-free medium to facilitate DNA absorption, and then the serum level was restored to 10% by addition of medium with 20% fetal bovine serum. Samples were collected by fixation in 3% formaldehyde at 37°C between 30 and 52 h after transfection. Formaldehyde was made up fresh from paraformaldehyde in phosphate-buffered saline (PBS) as previously described [70].

In some experiments, cells were treated with phorbol 12-myristate 13 acetate (PMA) and LPA (1-oleoyl-*sn*-glycero-3-phosphate) purchased from Sigma-Aldrich (St. Louis, Missouri) and Fluka (Buchs, Germany), respectively. Filamentous actin was stained with tetramethylrhodamine-isothiocyanate-(TRITC-) labeled phalloidin (Sigma-Aldrich), which was made up as a 0.4 *μ*g/mL stock solution and further diluted 1 : 600 in PBS. In some experiments, cells were stained with coumarin phallacidin (Invitrogen) made up at the stock concentration and further diluted in 5% goat serum before being diluted 1 : 150 for use. Samples were mounted with DAKO Cytomation mounting medium or Immuno-Fluore medium (MP Biomedicals, Solon, OH).

### 2.2. Epifluorescence Microscopy

**Figure 1:**
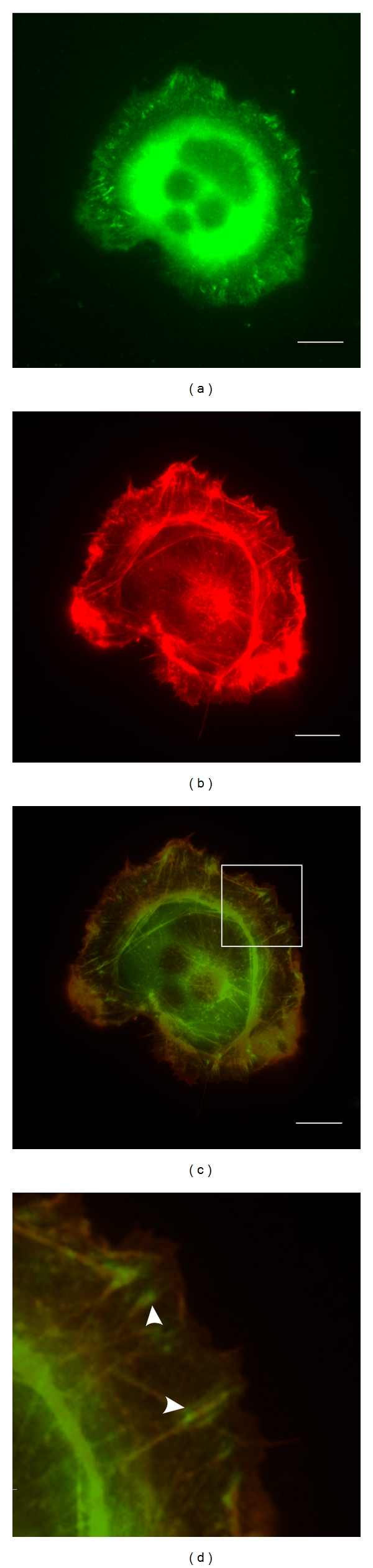
Representative micrographs of 1000 W cells transfected with GFP paxillin and stained with coumarin phallacidin. (a) Cell expressing GFP paxillin, (b) phallacidin on actin filaments and (c) merged frames of (a) and (b) (d), magnified region of frame (c) showing some of the FCs coinciding with the actin filaments (arrowheads) and some without any association. Bar = 10 *μ*m.

Cells expressing GFP paxillin were selected at random in the Zeiss Axiophot microscope equipped with 63x and 100x Neofluar lenses. Images were acquired with a Hamamatsu camera and Macintosh computer running OpenLab software (PerkinElmer, Seer Green, UK). For colocalization of signals, images were also acquired with the filter set used for DAPI or rhodamine, as well as in the green to obtain the GFP paxillin and phalloidin images. Thereafter, the images of both the actin-stained (Figure 1(a)) and the GFP paxillin (Figure 1(b)) frames of a single cell were processed in Adobe Photoshop CS3 extended version 10.0.1. The frames were then merged (Figure 1(c)), and the brightness and contrast was adjusted in separate layers of the image to identify the FCs associated with actin and those that were by themselves and not associated with actin.

For stress fiber counts, preparations stained with TRITC phalloidin were examined with a 40x Neofluar lens and rhodamine filter set, as described above. Positive cells were defined as those showing one or more robust fibers positioned centrally in the cell or crossing a central portion of the cell. Only single cells were tallied. At least 100 total cells were counted per sample [71].

### 2.3. Plasmids

DNAs with HA or FLAG tags were engineered in pXJ40 vector containing cytomegalovirus enhancer and promoter sequences [72, 73]. Enhanced green fluorescent protein (GFP) fused in frame to paxillin, T19N mutant of RhoA, wild-type PAK1 (wt-PAK), PAK^155−207^ domain binding PIX (PAK-interacting exchange factor), or kinase inhibitory domain (PAK^83−149^) of PAK1 were inserted in pXJ40. Nck1 was engineered in pGEX plasmid [59]. When more than one plasmid was introduced, the level of transfected agents was adjusted to give 0.5–1.0 *μ*g of transfected DNA in each chamber. Previously, we had found that Nck1 augmented large strap-shaped and triangular protrusions indexed by factor 7 values. Datasets no. 1 and no. 2 included cells transfected with the plasmids listed above. We also found two combinations of plasmids that inhibited filopodia and, to a lesser extent, large protrusions: (1) RhoA (T19N), Cdc42 (G12V), Rac1 (G12V) with PAK1 and (2) RhoA (T19N), *α*1-chimerin, and PAK1 [59]. These were used to ensure that the features of interest were present in some samples and lacking from others.

### 2.4. Image Processing for FCs

**Figure 2:**
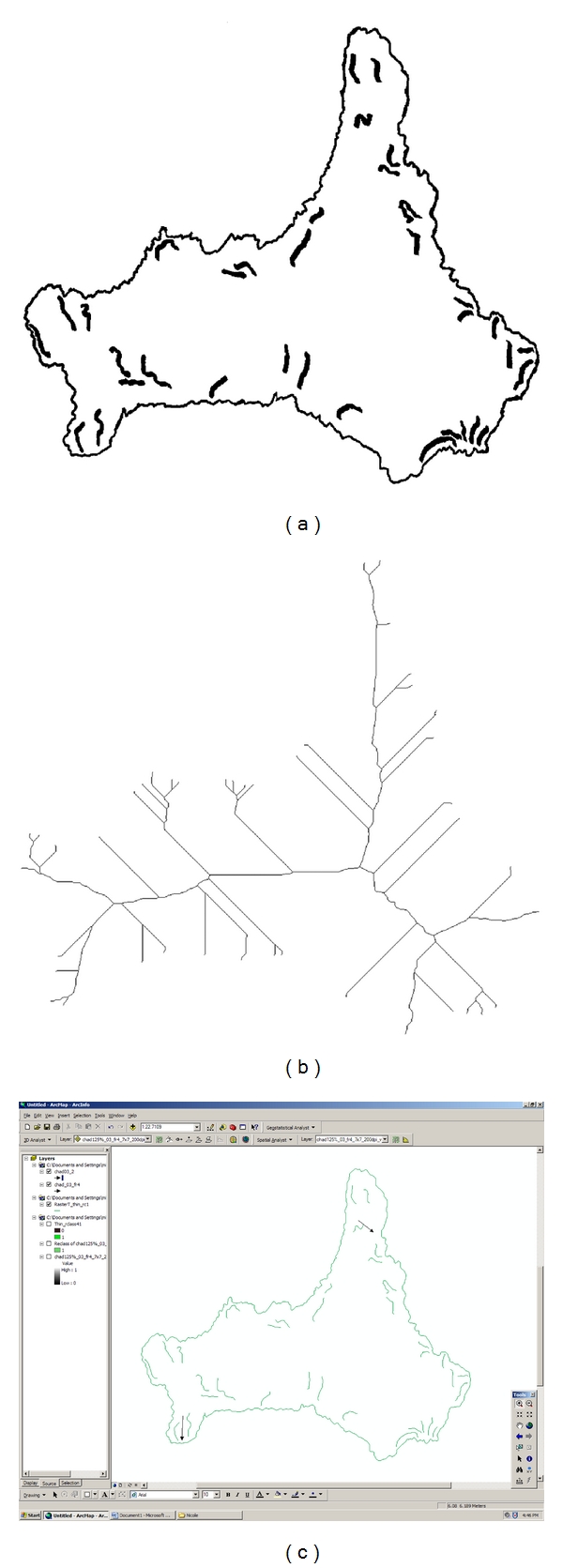
Image of tracings derived from one micrograph. (a) Scanned tracing of boundary from a representative cell and the FCs within it. (b) Skeletonized image of the cell in (a). (c) Linear directional mean analysis in ArcGIS. Arrows indicate mean direction of FCs within a protrusion.

For FC localization experiments, the cell edge and the FCs were traced on transparencies on a Dell computer running Adobe Photoshop 7 (Figure 2(a)). Images showing FC distribution were input into an IBM PC running MetaMorph software version 4.6r5 (Molecular Devices, Sunnyvale, CA). After applying a calibration, we evaluated FCs using the Integrated Morphometry Analysis module. The values of size and shape variables for each FC, as well as the *x*, *y* coordinates of its centroid, and its orientation to the horizontal axis were also determined. Some elliptical Fourier analysis (EFA) variables were also read out. In total, 114 variables were generated for each FC. Definitions of the Integrated Morphometry Analysis variables are available at site: http://support.meta.moleculardevices.com/docs/mm%20bag.pdf. For a different dataset, the cell edge and the two populations of FCs (see “Epifluorescence microscopy”) were traced separately on transparencies on a Dell microcomputer, as described above.

### 2.5. Generation of Factor 4, 5, 7, and 16 Values

TIFF images of traced cell edges were transferred to an SGI INDY FTP server (elvis.bgsu.edu), where contour extraction was performed and the primary shape variables were solved. The results were autoscaled and the variables converted to values of factors 4, 5, 7, and 16 by programs in C++ as previously described [59, 70].

### 2.6. Statistical Analysis

Variables from Metamorph software were analyzed by Pearson's correlation coefficient (PCC) in SAS [74] to determine whether they were related to factor values. For some data sets, the distribution of FCs by length/breadth ratio was analyzed. This is referred to below as “ellipticity.” Statistical analyses such as Student's *t*-test were done using Microsoft Excel 2007 and Minitab (State College, PA).

### 2.7. Spatial Statistical Studies with ArcGIS

To compare the angle formed by FCs with the long axis of the protrusion, we used linear directional mean extraction with ArcGIS to get the former and a procedure in Image J to compute the latter. The edge images were uploaded into Image J Launcher 1.41 u and the skeleton image made using the plug-in process called Skeletonize (Figure 2(b)). Cells without protrusions were selected as controls. The skeletonized images were loaded into Adobe Photoshop CS3 extended version 10.0.1, and the corresponding tracings with FCs were superimposed. These images were uploaded into the ArcGIS9 ArcMap version 9.2. FCs along the gradually curved edge (for controls) or along the edge of predetermined protrusions (subjects) were selected with the cursor (Figure 2(c)). The FCs were converted to polylines since the input feature has to be a polyline. Thereafter, a projection system was assigned, namely, World Geodetic System 1984 (WGS), which serves as a framework for locational measurement. This system would use the cell's center of mass as the origin and, accordingly, determine the angles of the selected FCs. The angle of the skeletal line projecting into the outermost edge of each protrusion was determined, and, in each case, the angle of FCs was subtracted from the skeleton angle. The absolute value of the difference between the FC angle and the skeletal line was used for further statistical analysis.

More information on spatial analysis by ArcGIS is available at http://webhelp.esri.com/arcgisdesktop/9.2/index.cfm?tocVisable=1&id=1922&pid=1919&topicname=Linear%20Directional%20Mean%20(Spatial%20Statistics)&pid=1919.

### 2.8. Myosin Inhibitor Treatments, Scanning Electron Microscopy, Cell Tracings, and Image Analysis

For myosin experiments, inhibitors to myosin II were added directly to the tissue culture medium for a treatment time of 2 hours, during the eighth hour of treatment with PMA and LPA. The inhibitors were used at their IC50 concentration and also at a 1 : 4 dilution. Blebbistatin (0.2 *μ*M and 0.8 *μ*M), Rho-kinase (ROCK) inhibitor H1152p (5.02 *μ*M and 20.2 *μ*M), MLC kinase inhibitors, K252a (6 nM and 24 nM), and SPC 16524 (170 *μ*M and 680 *μ*M) were used.

After treatment with the inhibitors, cells were fixed with 3% glutaraldehyde in 0.1 M phosphate buffer (pH 7.2) and exposed to an osmium tetroxide—thiocarbohydrazide—osmium tetroxide protocol to enhance the conductivity of the sample. Samples were dehydrated through a series of ethanol solutions, dried in a Samadri-780A critical point dryer, coated with a 2-nm Au-Pd layer in a Polaron VI-A sputter coater, and then imaged in a Hitachi 2700S electron microscope. Images of randomly selected cells were recorded on a Seikosha VP-3500 video printer. A tracing was made onto a transparency to record these edges. Tracings were scanned and converted into binary images using a HP Photoshop 3210 scanner. Contour extraction was performed in anonymous FTP server, elvis.bgsu.edu, using programs to solve shape analysis variables. The results were autoscaled by use of a C++ program, ‘‘autoscal.cxx.” The primary shape variables were converted to values of factors 4, 5, 7, and 16 by the program ‘‘cmpute_f7corr_scoring.cxx.”

## 3. Results

### 3.1. FC Characteristics and Protrusions

Our first objective was to establish whether FC shape and size characteristics were related to factor 7 protrusions or other quantifiable protrusions. Treatments known to increase factor 7 values [59, 70] were used. Cells were treated with PMA and LPA for 10 h to enhance the values or else transfected with the relevant PAK and Nck DNA constructs. Controls were treated with solvent vehicle alone and/or GFP paxillin plasmid. Images showing GFP paxillin fluorescence were analyzed in MetaMorph software to generate 114 variables related to the size and shape of the FCs (see Materials and Methods). Since there was considerable redundancy of these variables when applied to a one-bit image, 92 variables were analyzed by calculation of Pearson's correlation coefficient against the factor 7 value for each cell. Variables correlated with factor 7 values at a probability less than *P* value < 0.05 are itemized in Table 1. Pearson's correlation ranges from +1 to −1 and reflects the degree of linear relationship between two variables. A correlation of +1 indicates a perfect positive linear relationship, in other words, as *X* increases, *Y* also increases or vice versa. The opposite is true for a correlation of −1.

A number of dimensioned variables, such as area and perimeter, and variables reflecting the ellipsoidal shape of the FCs, namely, mean radius and equivalent radius, showed a statistically significant correlation with factor 7 values. The correlation coefficients were negative indicating that, as the values of the FC dimensions increased, there was a decrease in factor 7 value. For some of the variables, for example, fiber length and breadth, Metamorph software corrected for orientation relative to the *x*-axis. These variables were significantly correlated with factor 7 value (Table 1). Some of the variables, for example, width and height, quantified the size of FCs without making any corrections for their orientation but remained correlated. All variables correlated at a *P* value < 0.025 (97.5% confidence level) are shown in Table 1.

The above results showed that smaller FCs favored the generation of factor 7 features. This outcome was confirmed when the data collection was repeated on a second experimental dataset (data not shown). It was possible, although unlikely based on previous studies (see Section 1), that this was a property of other features, measured by factors 4, 5, and 16 values. We explored this possibility by analyzing the relationship of these other factors to FC measures. As shown in Table 2, many of the same variables were positively correlated with values of factors 4 (filopodia) and 5 (lamellae). Correlations with significance levels of *P* < 0.025 (97.5% confidence level) are highlighted. Since the dimensions of FCs were increased in cells with high factor 4 and 5 values, the larger and presumably more stable FCs contributed positively to formation of these features. Therefore, the inverse relationship was unique to factor 7 protrusions. Although a negative correlation was found for factor 16 values with FC dimensions, none of the variables was significant at *P* < 0.025 (Table 2). 

### 3.2. Actin Integration into FCs in Relation to Different Protrusion Classes

**Figure 3:**
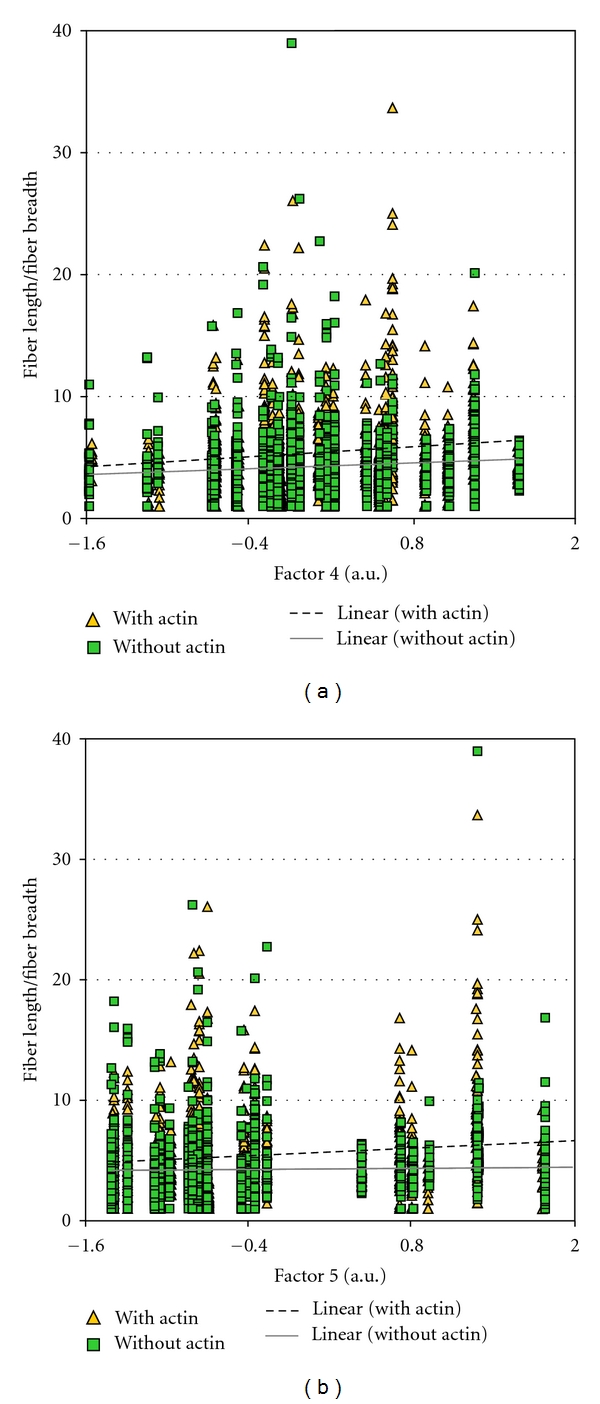
Plot showing the trend for with and without actin FC fiber length/fiber breadth versus factor values. (a) Factor 4. (b) Factor 5.

**Figure 4:**
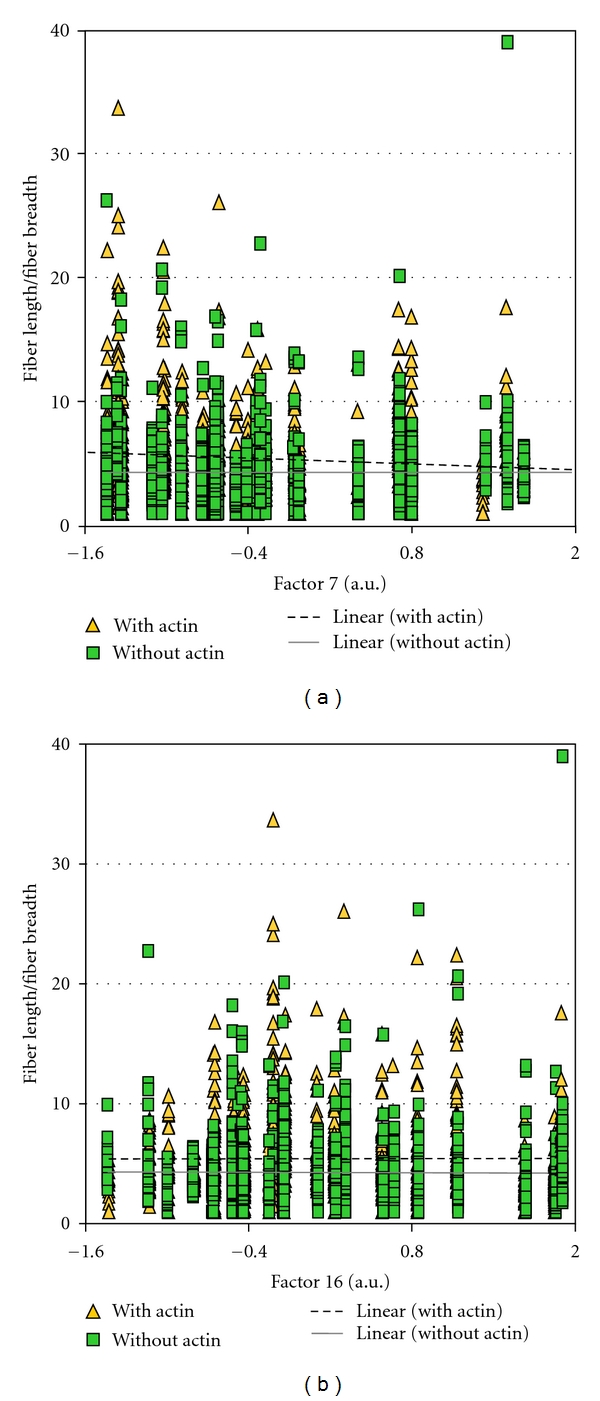
Plot showing the trend for with and without actin FC fiber length/fiber breadth versus factor values. (a) Factor 7. (b) Factor 16.

As stated in the Section 1, the larger and presumably more stable FCs are more likely to show integration with actin cables. In order to assess whether such integrated FCs were more highly correlated with factor 4 and 5, than with factor 7, we separated the FCs into classes that had no demonstrable attachment to actin and those that showed integration with cables. FCs differed in this experiment compared to the first experiment in that they showed greater variability in size and length (cf. Tables 1 and 3). For FCs without actin, the FC dimensions were negatively correlated with factor 7 values as indicated by PCC values significant with *P* values below 0.005 (highlighted on Table 3). There were two exceptions, however. Whereas the width measure originally showed an inverse relationship to factor 7 values, width became uncorrelated. This was also observed for the centroid *X* values for FCs without actin cables (Table 3). The other characteristic property of the larger FCs was that they were elongated. To visualize this relationship, the values for factors 4, 5, 7, and 16 were plotted against FC ellipticity. The with-actin category showed a positive relationship between the ellipticity of the FC and the values of factor 4 and factor 5 (Figure 3). The category of FC without actin integration did the same for factor 4 (Figure 3(a)) but not for factor 5 (Figure 3(b)). This confirmed the expectation that the FCs which were elongated and integrated with actin cables were correlated with these features because both were correlated with FC size. For factor 7 values, as expected from Table 3, there was a negative relationship to most dimensions of FCs regardless of their category. This was more pronounced for the with-actin category than the without-actin category (Figure 4(a)). There was no apparent regression of any FC dimension with values of factor 16 (Figure 4(b)). 

**Figure 5:**
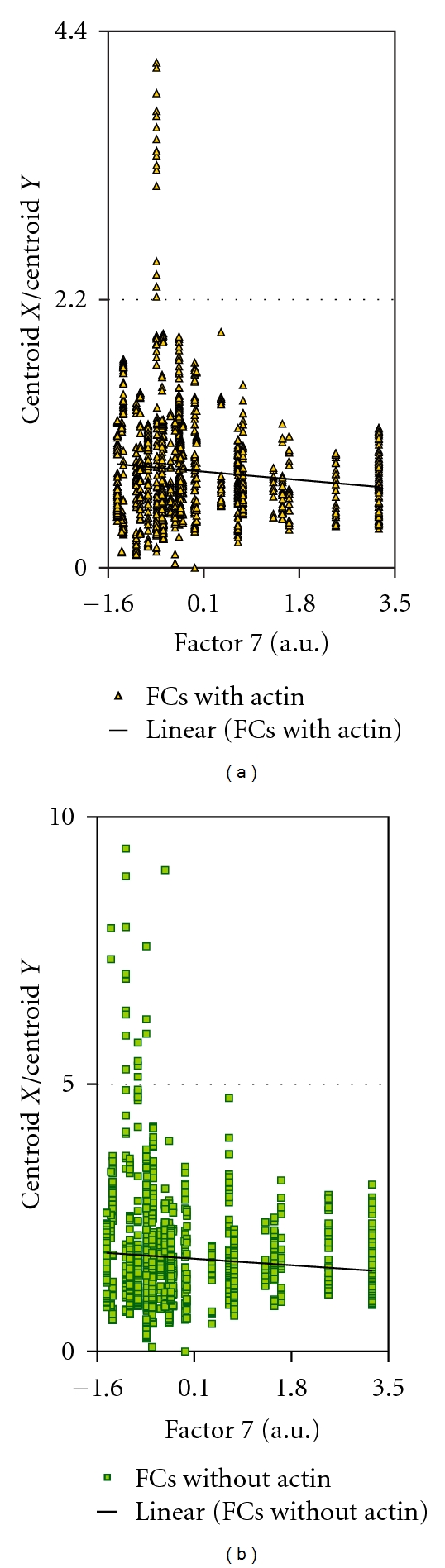
Ratio of the centroid *X*/centroid *Y* values for two classes of FCs plotted against factor 7 values.

**Figure 6:**
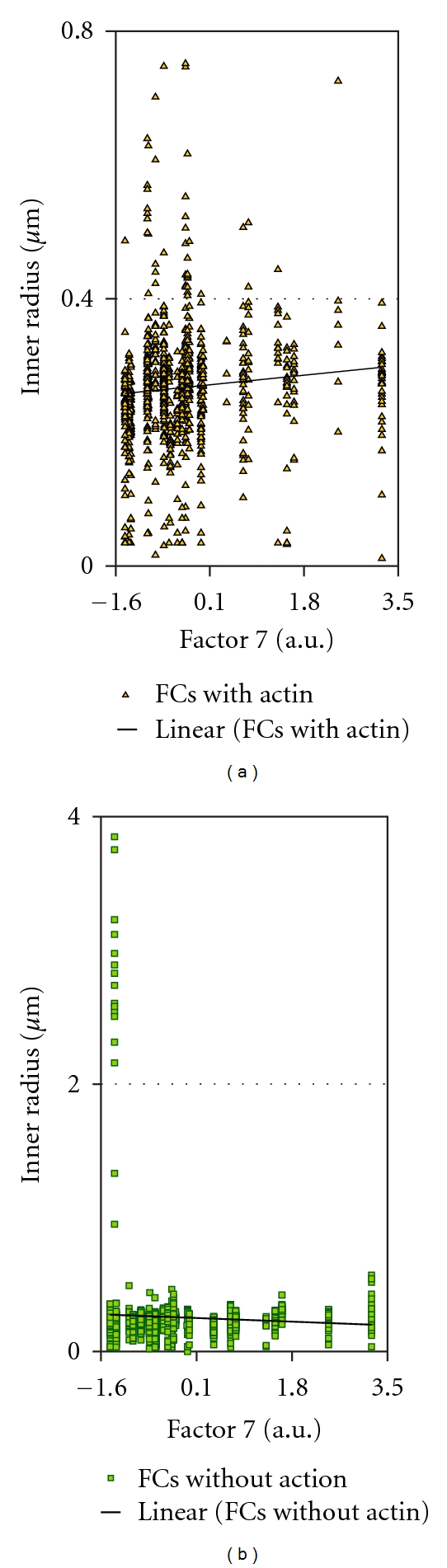
Plot showing the trends for the inner radius of the two classes of FCs with increasing or decreasing factor 7 values.

**Figure 7:**
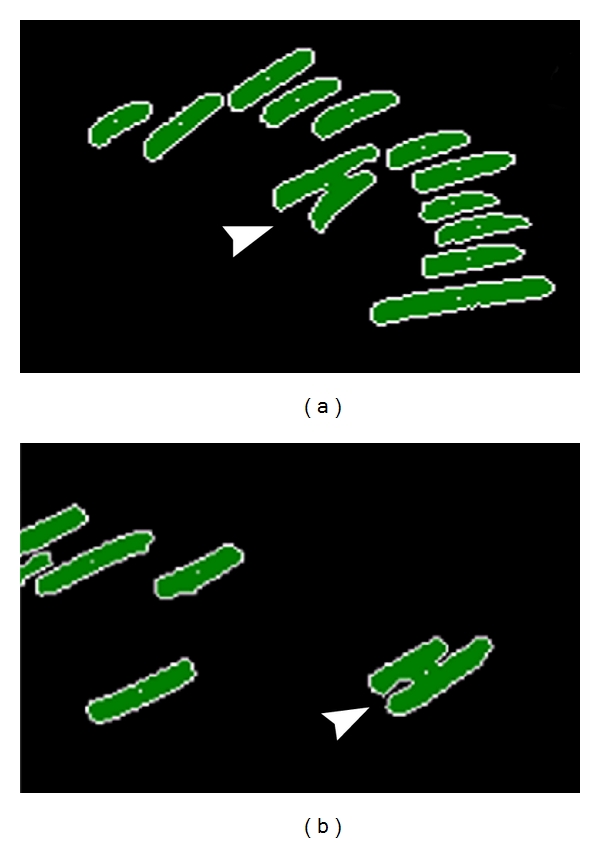
MetaMorph image showing representative FCs. Those with a large width compared to their length are designated (arrowheads). They were overlapping in the image due to converging angles of orientation (a) or lying at too close a distance to be resolved separately (b).

Remarkably, for the category of FCs with actin integration, two variables, namely, the inner radius and fiber breadth, were positively correlated with factor 7 values. Since both showed an inversion of the normal sign of the PCC (Pearson coefficient), the usual relationship between FC dimensions and factor 7 value was reversed for these FCs. Moreover, these PCCs were highly significant (Table 3). That the PCC for fiber breadth showed either a negative or positive sign, depending on the category of actin integration with the FC, suggested that there was a population of more stable FCs supporting the formation of factor 7 features. This finding explained the low absolute value of the correlation coefficients with factor 7, as both negative and positive relationships were rolled into the overall correlation. As FC length and outer radius were uncorrelated with factor 7, the FCs in this new category were small in all dimensions except width. This was confirmed by directly plotting the factor 7 values against the relative dimensions of FCs in the two classes. The raw values of centroid coordinates in the *X* and *Y* dimensions were plotted, because no external data were entered to normalize these values in the MetaMorph software (Figures 5 and 6). Because FCs with a fat contour were rare in the processed black-and-white images, we further explored the data by selecting FCs in the with-actin category that showed low ellipticity measurements. The results suggest that the FCs with dimensions positively correlated with factor 7 values are FCs with different orientations as shown in Figure 7.

### 3.3. Orientation

The above results suggested that the factor 7 feature was mainly attached to the substrata by point contacts but also required one or more stable FCs integrated with actin cables. Moreover, factors 4 and 5 were similar in being linearly related to FC size, except for one variable representing the angle made by the longest chord through the FC with the horizontal axis. Called orientation, this variable was correlated with values of factors 4 and 7 (Table 1) but not factor 5 (data not shown). The orientation differences were analyzed using another software package (Table 4). A mean difference of approximately 10 degrees was observed, regardless of whether the FCs were in the with-actin or without-actin category. 

### 3.4. Role of Myosin in the Factor Feature Formations

Perhaps the results suggested that slender actin stress fibers facilitated the formation or maintenance of factor 7 features. However, there is a consensus that some stress fibers are not contractile (see Section 4). To determine whether myosin activity was required to form or maintain the features, we treated cells with myosin inhibitors with different mechanisms of action. The cells were exposed to PMA and LPA for 10 h with addition of the myosin inhibitor at levels corresponding to the reported IC50, as well as one-third the IC50, for the final 2 h or treatment. When the values of factor 7 were solved, we found that only the Rho kinase inhibitor, H1152p, affected feature production (Table 5). Because the dissolution of stress fibers was a well-known effect of Rho kinase inhibitors, the result was interpreted as meaning stress fiber production might inhibit feature formation. When we assayed the frequency of stress fiber-containing cells, the data confirmed the potent effect of H1152p inhibition on stress fiber formation. This suggested that H1152p enhanced factor 7, because the stress fibers exerted centripetal contractile forces on the cells and made it more difficult for them to extend protrusions. Data showing a modest effect on factor 7 of the MLC kinase inhibitor, which also prevented stress fiber formation, was consistent with this interpretation.

**Figure 8:**
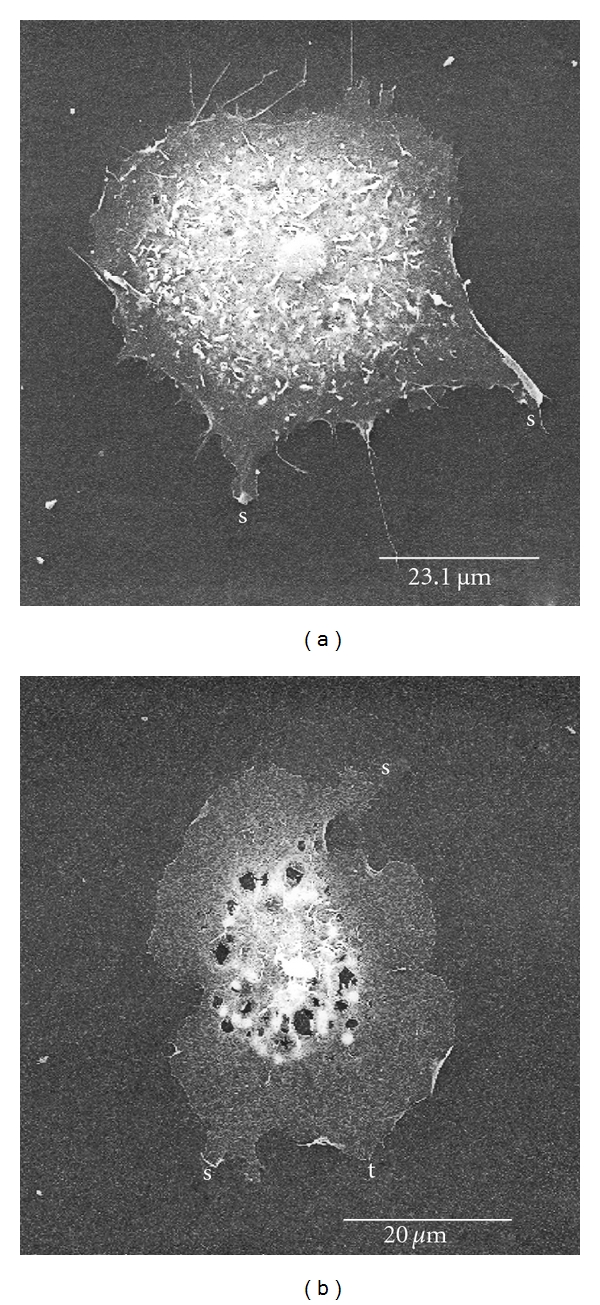
Representative SEM micrographs of cells showing the effects of myosin inhibitor (blebbistatin) and the Rho-kinase inhibitor (H1152p) treatments on factor 5 and factor 7 values. (a) Cell treated with blebbistatin showing a factor 7 value of 2.29 and a low factor 5 value of 0.039. (b) Cell treated with H1152p with a factor 7 value of 2.39 and a high factor 5 value of 3.88. Factor 7 features on the cells are represented by t: triangular-shaped and s: strap-shaped structures.

According to the above interpretation, it was anticipated that other protrusions, in addition to factor 7, might respond similarly to myosin inhibitors. The effect of H1152p on factor 5 values was the same but the features were eliminated by blebbistatin (Table 6), which uncouples ATPase and thereby removes the myosin from actin. Since both factor 7 and factor 5 represent large, bulky features, we wanted to investigate the difference introduced by blebbistatin treatment. Blebbistatin had no effect on factor 7 features. Cells in the H1152p and blebbistatin treatment groups included examples showing high values for both protrusions and low values only for factor 5 (Figure 8). The cell shown, which was high in both values, exhibited both protrusions and invaginations thereby displacing much more mass from the cell center. For factor 4 values, only the MLC kinase inhibitor had an effect, and it knocked down the features at both concentrations (Table 7).

**Figure 9:**
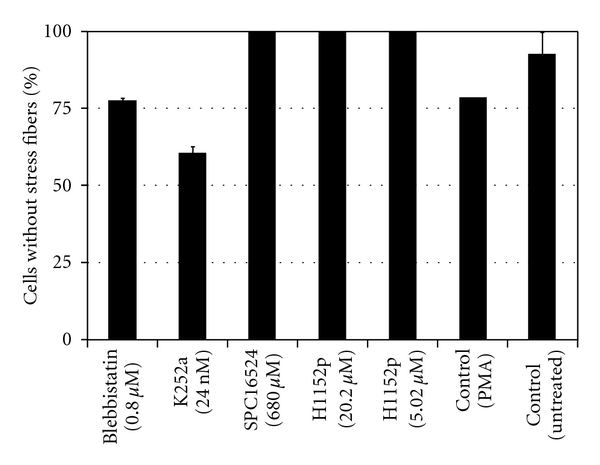
Cells lacking stress fibers after treatment with myosin II inhibitors in comparison to control. Cells were treated with PMA for 10 h to induce stress fiber formation. Stress fiber-containing cells were determined as described in Section 2.

Because the reversal of stress fibers was a well-known effect of myosin inhibitors, the above results suggest that neither peripheral nor central myosin-mediated contractile cables facilitates factor 5. When we assayed the frequency of stress fiber-containing cells (Figure 9), the data confirmed that both ROCK and MLC kinase inhibitors led to stress fiber dissolution. This supported the interpretation that the stress fibers exerted centripetal contractile forces on the cells and made it more difficult for them to extend protrusions. Since both protrusions were resistant to the effects of MLC phosphorylation inhibitors, it was unlikely that stress fiber dissolution alone accounted for the formation or extension of the features. 

## 4. Discussion

### 4.1. FC Properties Distinguishing Factors from One Another

Since the dimensioned variables were negatively correlated with factor 7 values, smaller FCs must have favored the generation of the features. This suggested that the feature represented by factor 7 had FCs that, in other types of cells, were subject to differential regulation by the Rho-family GTPases. These GTPases are implicated in pathways that dictate contact initiation, maturation, and turnover. Neuronal growth cones were found to have point contacts [29], which were regulated by coordinated Rho and Rac GTPase activity to stabilize membrane protrusions and neurite outgrowth [30]. In nonneuronal cells, such small FCs were called punctate FCs [31] or Rac-induced FCs [32]. The current results suggest that the factor 7 feature is equivalent to a neurite primitive structure in the nonneuronal cell. The enhancement of factor 7 features with cytochalasin D treatment offered indirect support for this conclusion (data not shown), since microtubules are well known to be advanced further in neurites upon depolymerization of the actin cytoskeleton [75]. It is also consistent with the sensitivity of factor 7 features to lower temperatures [76], which is known to selectively destroy microtubule integrity. 

The requirement for punctate FCs was unique to cells with high factor 7 values, as the values of factors 4, 5, and 16 failed to show negative correlations with FC dimensions. On the contrary, factors 4 and 5 showed significant positive correlations with FC dimensions. Many studies showed that, upon maturation, FCs became elongated [38, 44, 77, 78]. Thus, it was predictable that the ratio of FC length to breadth would show positive correlations with the values of factors that were dependent upon mature FCs. Rho activity causes the formation of long (2–5 *μ*m^2^), dash-shaped FCs associated with actin stress fibers [64, 77], reviewed in [79, 80]. The maturation process occurs by immature FCs recruiting actin filaments to become large, elongated focal adhesions [81]; reviewed in [37, 82]. Thus, both mature FCs and bundling of actin filaments are augmented by RhoA activity. To recruit actin stress fibers on the cytoplasmic surface of the FC site, the two structures are thought to become associated through actin-binding proteins such as talin and vinculin [81]. Since the MLC kinase inhibitors caused stress fiber dissolution but failed to enhance protrusion formation, dissolution alone was insufficient for the formation or extension of the features. Rather, a balance of pathways, both central and peripheral, may be affected by the Rho kinase inhibitor, H1152p.

When the significance of correlations of FC length and width was analyzed for FCs with actin and without actin, the highest *F*-values were observed for factors 4 and 5 in the category of with-actin FCs. The *F*-value for FCs in the without-actin category was also high for factor 4 but negligible for factor 5. Thus, the factor 5 features had a greater dependence on large, elongated structures, that is, FCs and cables, than any other class of protrusion. The absence of any significant relationship between factor 5 values and the without-actin category of FCs suggested that these features required anchors integrated with stress fibers (see below). The distinct relationships of FCs analyzed here, that is, with-actin and without-actin, are related to concepts of maturation of FCs. The best maturation model was consistent with the inherent directionality of actin integration into the FC [83] and was based on a postulated mechanism favoring elongation over sidewise aggregation of constituents. Factor 4 only showed a correlation between factor values and the without-actin category of FCs, so FC maturation was not relevant to filopodia formation. Factor 7 relationships to the FC, however, are clearly inconsistent with the theoretical model. That the inner radius and width of the with-actin category of FCs were positively related to factor values suggested that FCs may have been compact in shape. This was supported by an absence of correlation with the mean radius or outer radius, along with a lack of regression on FC length/breadth. 

### 4.2. Differences in Global Organization of FCs

ROCK enhances the phosphorylation level of MLC cooperatively by two mechanisms. First, it phosphorylates the myosin binding subunit (MBS) of myosin phosphatase. Myosin phosphatase is composed of a 37-kD phosphatase catalytic subunit and the 20-kD MBS regulatory subunit [84, 85]. By phosphorylating MBS, ROCK dissociates the subunits and prevents dephosphorylation of MLC, thereby prolonging the activation of myosin. Secondly, ROCK phosphorylates MLC at the same site that is phosphorylated by MLC kinase, thereby stimulating myosin ATPase activity [46].

The system of actin stress fibers in the cell included some which were associated with an FC at either end, while others had an FC at only one end or were independent of FC association [80]. ROCK and MLC kinase may play distinct roles in regulating MLC phosphorylation and subsequent myosin II activation in these two cable systems. With ROCK inhibition, MLC kinase-activated stress fibers are not assembled in the cell center [86]. The physiological function of actin cables at the periphery of the cell also appeared to differ from that of the central cables [67, 87, 88]. In mouse embryonic fibroblasts, FCs showed the same periodic localization as MLC kinase suggesting that early adhesion-site formation is designated by myosin-dependent retractions [89]. Moreover, MLC kinase activity was implicated in periodic contractions coinciding with actin transport in the lamellipodium and with lamellipodium retraction [23]. The MLC kinase inhibitor, unlike the ROCK inhibitor, failed to affect the contractility of actin cables [90]. 

To determine whether myosin activity was required to form or maintain the features, we exposed cells to PMA and LPA for 10 h with addition of the myosin inhibitor at levels corresponding to the reported IC50, as described in the Section 2. Only the Rho kinase inhibitor, H1152p, had an effect on factor 7 values (Table 5). Surprisingly, H1152p enhanced feature production, whereas we had expected to find a decrease in factor 7 values. The different effects of kinase inhibitors on factor 7 is consistent with the existence of separate cable systems with different levels of contractility. We reported previously that factor 7 values declined with an increase in stress fibers [70], so that H1152p-mediated enhancement may be caused by the release of isometric tension placed on the cytoplasm by the central cables. Since the expression of dominant active ROCK caused neurite retraction in neuronal cells [47, 91, 92] and ROCK limited protrusions in nonneuronal cells [93], the H1152p effect is consistent with the identification of the factor 7 feature as a neurite primitive. Previous findings also lent support to this characterization of factor 7, because destabilizing the actin cytoskeleton locally led to a neurite's being designated for development as an axon [94]. The directional preferences invoked in guidance and chemotaxis are dependent upon a local pattern, which is subject to self-destabilization. The latter process in turn may rely on travelling waves or oscillations [95], accounting for the periodic distribution of MLC kinase in the lamellipodium. Because of the lack of any guidance or chemotactic stimulus in the culture environment, we consider the emergence of the factor 7 features to represent a mechanism of pattern formation for protrusion extension. 

The most surprising result observed was the increase in factor 5 values with myosin inhibitors. Both H1152p and SPC16524 increased the values of factor 5. This is consistent with previous reports that myosin II is inessential for protrusions [42, 48]. On the other hand, myosin in protrusions such as lamellipodia was thought to drag actin filaments in from the cell edge by moving them past antiparallel filaments from the lamella [21]. 

The above results indicated that actomyosin contractility plays a negligible role in the protrusions, with the exception of filopodia (see below). The effects of ROCK inhibitors must be interpreted with care, however. Some of the other substrates of ROCK, adducin [96, 97], and myristoylated alanine-rich C kinase substrate (MARCKS) [98] are also protein kinase C substrates. However, it is known that H1152p enhanced ruffling [96, 97], which has been shown to be closely linked to an increase of factor 5 in previous studies [57]. Therefore, enhancement of these protrusions by myosin inhibitors may be attributed to dual mechanisms, one being the release of the isometric tension exerted by actomyosin contraction downstream of Rho activation and the second ruffling enhancement. As expected, factor 4 values were the same in the PMA-treated and untreated control samples. The values were decreased by both the concentrations of MLC kinase inhibitor (SPC16524) but not elevated by any of the myosin inhibitors (Table 7). This result was unsurprising, because filopodia (factor 4 values) were readily decreased but difficult to enhance [59, 99].

The current research shows that the origin of various features can be studied by the new approach of classifying cell features on a quantitative and qualitative basis. This enables a correlation between morphology and physiological regulation that has not hitherto been possible. Findings reported in this set of experiments include the following. The feature represented by factor 7 is a neurite primitive with points of resemblance to the nascent axon. The factor 5 feature, which represents mass distribution away from the cell center, is not reliant upon myosin II to advance the cytoplasm in a net outward direction. Thirdly, the features are all regulated differently as indicated by their relationship to FC dimensions and ellipticity. 

## Figures and Tables

**Table 1:** Dimensioned and raw variables whose values are correlated with factor 7 values^a^.

Variable	Mean (*μ*m)^b^	Standard deviation (*μ*m)^b^	PCC^c^	*P* value
Dimensioned variables				
Area	4.56	26.07	−0.085	0.015
Perimeter	6.90	9.60	−0.090	0.009
Mean radius	0.72	0.94	−0.090	0.001
Equivalent radius	0.72	0.95	−0.091	0.009
Width	1.86	2.67	−0.088	0.011
Height	1.70	2.18	−0.089	0.010
Fiber length	2.77	4.10	−0.087	0.013
Fiber breadth	0.67	0.92	−0.089	0.011
Orientation	−0.71	47.00	−0.080	0.023

Raw (nondimensioned) variables				
Centroid *Y *	62.33	115.24	−0.089	0.011
Centroid *X *	41.89	73.35	−0.104	0.003

^a^Number of samples = 820; number of cells = 37.

^b^Units are *μ*m^2^ for area and degrees for orientation.

^c^PCC: Pearson's correlation coefficient.

**Table 2:** Dimensioned and raw variables whose values are correlated with factor 4, 5, or 16 values^a^.

Variable	Dimensions		*Factor 4*		*Factor 5*		*Factor 16*	
	Mean (*μ*m)^b^	Standard deviation (*μ*m)^b^	PCC	*P* value	PCC	*P* value	PCC	*P* value
Area	4.28	31.67	0.077	0.0003	0.102	<0.000	−0.034	0.116
Perimeter	7.63	10.32	0.108	<0.000	0.122	<0.000	−0.028	0.192
Mean radius	0.92	1.15	0.112	<0.000	0.132	<0.000	−0.034	0.110
Equivalent radius	0.77	0.87	0.103	<0.000	0.120	<0.000	−0.031	0.149
Width	2.15	1.34	0.073	0.000	0.079	0.000	0.026	0.223
Height	1.97	1.36	0.107	<0.000	0.092	<0.000	−0.047	0.029
Fiber length	3.16	4.68	0.110	<0.000	0.123	<0.000	−0.028	0.196
Fiber breadth	0.65	0.56	0.079	0.000	0.098	<0.000	−0.026	0.227
Centroid *Y *	94.5	88.5	0.086	<0.000	0.088	<0.000	−0.008	0.723
Centroid *X *	67.83	86.8	0.033	0.125	0.066	0.002	−0.022	0.315

^a^Number of samples = 2170; number of cells = 37.

^b^Units are *μ*m^2^ for area except centroid values which are integer values without units.

**Table 3:** Dimensioned and raw variables generated for FCs with actin and without actin showing correlation with factor 7^a^.

Variable	*FCs without actin* (*n* = 1252)				*FCs with actin* (*n* = 918)			
	Mean (*μ*m)^b^	Standard deviation (*μ*m)^b^	PCC	*P* value	Mean (*μ*m)^b^	Standard deviation (*μ*m)^b^	PCC	*P* value
Area	5.72	41.62	−0.107	0.000	2.32	1.75	0.032	0.327
Inner radius	0.25	0.30	−0.065	0.021	0.27	0.09	0.156	<0.000
Outer radius	1.52	2.65	−0.079	0.005	1.76	1.009	−0.011	0.740
Mean radius	0.87	1.45	−0.082	0.004	0.99	0.50	−0.003	0.933
Equivalent radius	0.75	1.12	−0.085	0.003	0.81	0.28	0.035	0.294
Width	2.24	3.70	0.053	0.060	2.50	1.63	−0.092	0.005
Height	2.08	4.30	0.134	<0.000	2.40	1.63	−0.040	0.234
Fiber length	2.94	5.82	−0.082	0.005	3.47	2.32	−0.041	0.210
Fiber breadth	0.65	0.73	−0.082	0.003	0.70	0.16	0.180	<0.000
Centroid *Y *	98.8	114.70	−0.096	0.000	88.62	23.04	−0.006	0.860
Centroid *X *	71.30	112.04	−0.094	0.000	63.16	25.72	−0.075	0.022

^a^Number of cells = 26.

^b^Units are *μ*m^2^ for area except centroid values which are integer values without units.

**Table 4:** Two sample *t*-tests on the absolute difference of the mean angular differences between the long axis of the FC and the skeletons of the protrusions.

FCs with actin	Control group			Experimental group			*t*-value^b^	*P* value
	*N* ^ a^	Mean	Std Dev	*N*	Mean	Std Dev		
	33	14.33	9.7	67	24.35	25.05	−2.87	0.005

FCs without actin	Control group			Experimental group			*t*-value	*P* value
	*N*	Mean	Std Dev	*N*	Mean	Std Dev		
	30	12.66	10.77	85	22.67	24.58	−3.02	0.003

^a^Number of cells sampled.

^b^Value obtained in Student's *t*-test.

**Table 5:** Effects of Rho-kinase/myosin inhibitors on the prevalence of factor 7 features^a^.

Tukey grouping			Mean	Combination of agents
A			0.92	H1152p, ROCK inhibitor (20.2 *μ*M)
A			0.79	H1152p (5.0 *μ*M)
A	B		0.12	SPC16524, MLC kinase inhibitor (680 *μ*M)
	B		0.03	Control (PMA + LPA)
	B	C	−0.19	SPC16524 (170 *μ*M)
	B	C	−0.20	Blebbistatin, myosin ATPase inhibitor (0.2 *μ*M)
	B	C	−0.37	Blebbistatin (0.8 *μ*M)
		C	−0.85	Control (untreated)

^a^Means with the same letter are statistically indistinguishable at the level *P* < 0.05.

**Table 6:** Effects of Rho kinase/myosin inhibitors on the prevalence of factor 5 features^a^.

Tukey grouping					Mean	Combination of agents
A					0.830	H1152p, ROCK inhibitor (20.2 *μ*M)
A	B				0.525	SPC16524, MLC kinase inhibitor (680 *μ*M)
A	B	C			0.353	H1152p (5.02 *μ*M)
	B	C	D		0.072	Control (PMA + LPA)
		C	D	E	−0.195	SPC16524 (170 *μ*M)
		C	D	E	−0.371	Blebbistatin (0.8 *μ*M)
			D	E	−0.405	Control (untreated)
				E	−0.592	Blebbistatin (0.2 *μ*M)

^a^Means with the same letter are statistically indistinguishable at the level *P* < 0.05.

**Table 7:** Effects of Rho kinase/myosin inhibitors on the prevalence of factor 4 features^a^.

Tukey grouping			Mean	Combination of agents
A			0.599	H1152p (5.02 *μ*M)
A			0.359	Blebbistatin (0.2 *μ*M)
A			0.305	H1152p, ROCK inhibitor (20.2 *μ*M)
A			0.251	Control (PMA + LPA)
A			0.236	Control (untreated)
A	B		−0.064	Blebbistatin (0.8 *μ*M)
	B	C	−0.629	SPC16524, MLC kinase inhibitor (170 *μ*M)
		C	−0.882	SPC16524 (680 *μ*M)

^a^Means with the same letter are statistically indistinguishable at the level *P* < 0.05.
